# Study of the probiotic potential and evaluation of the survival rate of *Lactiplantibacillus plantarum* lyophilized as a function of cryoprotectant

**DOI:** 10.1038/s41598-021-98723-0

**Published:** 2021-09-27

**Authors:** Aida Yuste, Esteban Leonardo Arosemena, M. Àngels Calvo

**Affiliations:** Grupo de Microbiología Aplicada y Medioambiental, Facultad de Veterinaria, Universidad Autónoma de Barcelona, Bellaterra, Barcelona, Spain

**Keywords:** Microbiology, Health care

## Abstract

The benefits of probiotics for the improvement of animal health status have been of great interest in recent years. For this reason, in this study was aimed at assessing a strain with probiotic potential to be added to the feed. Therefore, the objective of this trial is to use a strain with probiotic potential isolated from the intestinal microbiota of *Helix aspersa* Müller to subsequently add it to the feed of this species to improve its health status. So, the strain is characterized, and its probiotic potential is demonstrated. Finally, with the aim of preserving the probiotic strain by freeze-drying so that it can later be added to the feed, different cryoprotectants were studied that could give it a higher survival rate over time. The cryoprotectant that gives the best result with strain survival rate is trehalose 15%.

## Introduction

The World Health Organization (WHO) in cooperation with Food and Agriculture Organization of the United Nations (FAO) define probiotics as living microorganisms that administered in adequate amounts confer a benefit on the host such as the reduction of enteric infections because they have a resistance to this pathogens^1–3^. In the last few years, the research field of probiotics has experienced a great boom allowing the development and commercialization of different products, being those that belong to the genus *Lactobacillus, Bifidobacterium,* and *Streptococcus* the most used, but there are many more genus that are included as possible probiotics^3^. To be considered a probiotic strain, its origin must be known, and it must be identified molecularly and phenotypically. In addition, it must meet a few guidelines^4^. Some of them are related to safety such as not being sensitive to antibiotics and not having pathogenicity. Also, they must also have several characteristics that provide benefits to the host including the presence of certain antimicrobial activity and be resistant to gastric juices and bile salts so that they can reach the host's intestine^5,6^. The bacteria used in this study is *Lactiplantibacillus plantarum* that was isolated from the snail microbiome because it’s important that it’s a bacterium that is normally present in its microbiota. So, that the intake of probiotic has a beneficial effect for the host, it must have an adequate number of cells in the intestine (ranged from 0 to 2 × 10^9^ CFU/g)^7^, which is why the preservation of microorganisms is necessary. In addition, these techniques also aim to keep the crop viable for a long period of time and with minimal genetic changes.

The lyophilization is a drying method that consists in the separation of water or other solvent separation through a freezing process and a subsequent sublimation of the ice under vacuum and low temperatures conditions, making it a dry product that can be stored at room temperature and the result is a product in powder form and can be mixed well with the feed. In addition, this method results in no growth because the water activity (a_w_) is zero and it is also a technique that allows maintenance of viability and purity over a long period of time^8,9^. To protect these bacteria from the damage caused by the freezing process a cryoprotectants is added^10^. There are many cryoprotectants such as skimmed milk or different carbohydrates (trehalose, glycerol, sucrose, among others)^8,9^. The cryoprotectants chosen to evaluate the survival rate provided to bacteria are trehalose, sucrose, skimmed milk, maltodextrin, and the combination of skimmed milk with sucrose. These cryoprotectants are classified according to their nature, trehalose and sucrose being of the disaccharide type, maltodextrin of the polysaccharide type and finally, skimmed milk is of the complex substrates type^11^. Skimmed milk, sucrose and trehalose are frequently used as cryoprotectants^10^. The concentration used depends on different studies and their results^12–15^.

The aim of this study is verifying the probiotic potential of the strain and the evaluation of its conservation with different cryoprotectants because the aim is to add it to the feed of *Helix aspersa* Müller to improve its health status with a bacterium that is beneficial and is already part of its microbiota and it will not have any adaptation problems.

## Material and methods

The *L. plantarum* strain previously isolated in the Applied and Environmental Microbiology Research Group was preserved in cryovials Cryioinstant (Ref: 409113/6, Deltalab S.L., Spain) in the − 80 °C freezer. The microorganism was recovered by incubating two porous beads in 30 mL of MRS broth (Ref: BK070HA, Biokar Diagnostics, France) for 24 h at 37 °C in aerobic environment.

After the incubation period, a serial dilution was prepared up to the concentration of 10^–8^. From the dilution bank, MRS agar (Ref: BK089HA, Biokar Diagnostics, Francia) was seeded to establish the concentration of the microorganism and a blood agar (Ref: 254005, Becton Dickinson, Germany) was also seeded to evaluate its possible hemolytic capacity. Plates were incubated at 37 °C for 24 h, in aerobic environment.

So, to corroborate the absence of contamination throughout the preservation period, the strain was identified by phenotypic and molecular tests.

The tests carried out for phenotypical identification were Gram stain, spore stain and enzymatic tests of Catalase and Oxidase (Ref: 55 635, bioMérieux SA, France). The methodologies used have been the traditional in Microbiology^16^.

Then, an API 50 CH gallery (Ref: 50 300, bioMérieux SA, France) was inoculated with API 50 CHL Medium (Ref: 50 410, bioMérieux SA, France) to corroborate the identification of the strain.

### Whole genome sequencing (WGS)

Whole Genome Sequencing (WGS) using Oxford Nanopore Technology has been used for strain’s molecular identification.

6 mL of bacterial liquid culture overnight was used for DNA extraction with ZymoBIOMICS™ DNA Miniprep Kit (Zymo Research, Irvine, CA, USA). DNA quality and quantity were determined using Nanodrop 2000 Spectrophotometer and Qubit™ dsDNA BR Assay Kit (Fisher Scientific SL, Madrid, Spain).

A sequencing library was prepared using the Rapid Barcoding Sequencing kit (SQK-RBK004; Oxford Nanopore Technologies). The barcoded sample was loaded in a MinION FLO-MIN106 v9.4.1 flow cell and was sequenced in a MinION Mk1B for 22 h approximately. The fast5 files were basecalled with Guppy 4.0.11 (Oxford Nanopore Technologies) with high accuracy basecalling mode, demultiplexed and adapters trimmed using all the parameters by default.

Taxonomy was assigned using WIMP workflow from EPI2ME platform^17^. To be ensure about taxonomy classification, sequences were de novo assembled using Flye 2.8.3^18^ and the raw assembly was compared to *L. plantarum* strain SK151 chromosome (NZ_CP030105.1) by average nucleotide identity (ANI) using fastANI 1.32^19^.

### Tolerance to bile salts

The methodology used is a modification of Girmé^15^ and Vinderola and Reinheimer^20^. Four MRS broths were prepared with different concentrations of bile salts (Ref: B8756-10G, Sigma-Aldrich Co., USA): 0, 0.3%, 0.5% and 1%; and 2% of the study strain is added to each one. They are incubated at 37 °C for 180 min. After this time, three aliquots were taken from each sample to quantify absorbance at 560 nm and a dilution bank up to 10^–6^ was also made to seed it on MRS agar to make a count. The rest of the sample was incubated again until 24 h. After this time, the analyses were repeated with three aliquots again from each sample.

### Lysozyme tolerance

The methodology used is a modification of Girmé^15^. Four MRS broths were prepared with different lysozyme concentrations (Ref: L6876-1G, Sigma-Aldrich Co., USA): 0, 0.01%, 0.02% and 0.03%; and 2% of the study strain is added to each one. They were incubated at 37 °C for 60 min. After this time, three aliquots were taken from each sample to measure absorbance at 560 nm and a serial dilution was made until to − 6 to seed it to MRS agar to make a count. The rest of the sample was incubated again for up to 24 h. After this time, three aliquots from each sample were seeded again, and the absorbance was checked at 560 nm.

### Antibiotic sensitivity

**Figure 1:**
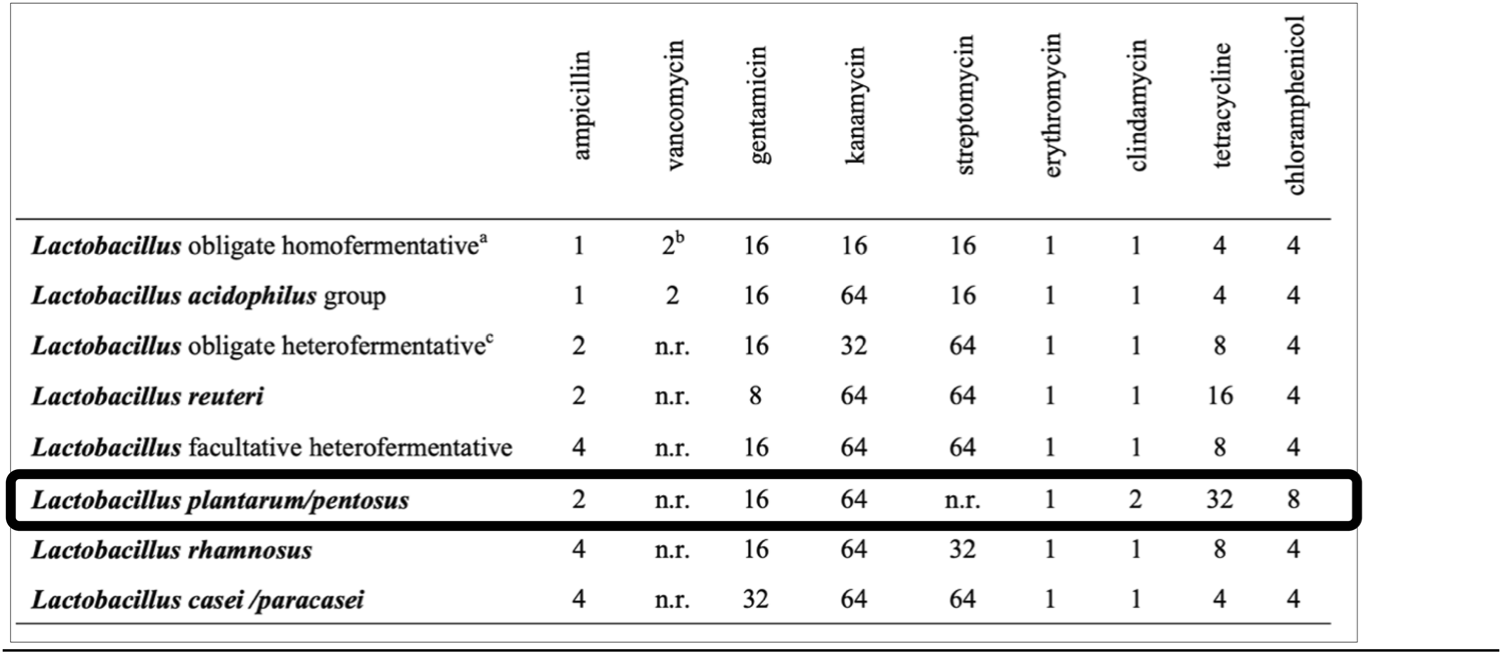
Antibiotics to test the safety of different strains with probiotic potential (EFSA, 2012)^21^. Values are expressed in mg/L; *n.r*. not required.

Two different methodologies were used to test the antibiotics required by the European Food Safety Authority (EFSA) to evaluate the safety of strains with probiotic potential (see Fig. 1)^21^. In this case, as it is a *L. plantarum* the antibiotics in powder form to be tested in the Broth dilution method are ampicillin (A9393-5G, Sigma-Aldrich Co, USA), gentamicin (G-3632, Sigma-Aldrich Co, USA), kanamycin (60615), Sigma-Aldrich Co, USA), erythromycin (E0774-5G, Sigma-Aldrich Co, USA), clindamycin (C5269-10MG, Sigma-Aldrich Co, USA), tetracycline (87128, Sigma-Aldrich Co, USA) and chloramphenicol (C0378-5G, Sigma-Aldrich Co, USA).

For the other test, the antibiotics were in disc form and were supplied by Becton Dickinson Sensi-DiscTM, Germany in the concentration indicated by the company.

1. Disk diffusion: A 0.5 McFarland suspension (Ref: 70 900, bioMérieux SA, France) of our strain was prepared and 100 $\mathrm{\mu L}$ were sown in Müller-Hinton agar (Ref: CM0337, Oxoid Ltd, United Kingdom). Two discs of the same antibiotic were placed on each plate. They were incubated for 24 h at 37 °C. Finally, the halo of inhibition was measured with a caliper.
2. Broth dilution method to determine the minimal inhibitory concentration (MIC): 96-well plates were used, as there were 7 antibiotics to be evaluated and 8 rows, the last row was empty as each row corresponds to a different antibiotic. 100 $\mathrm{\mu L}$ of Müller–Hinton broth (Ref: CM0405, Oxoid Ltd, United Kingdom) was dispensed in all wells. 2 × of the maximum antibiotic concentration was prepared and administered in the first column, homogenized well, and distributed 100 $\mathrm{\mu L}$ to column 2 and repeated up to column 10 where 100 $\mathrm{\mu L}$ are removed. A 0.5 McFarland suspension of our strain was prepared and 10 $\mathrm{\mu L}$ were inoculated into all the wells except column 12. It was incubated at 37 °C for 18 h. Once this time have been elapsed, it was read by an ELISA reader at 630 nm.

### Antimicrobial activity

The antimicrobial activity of this strain was tested against *Salmonella enterica* CECT 554*, Pseudomonas aeruginosa* CECT 119*, Listeria monocytogenes* CECT 4031*, Escherichia coli* CECT 434*, Klebsiella oxytoca* CECT 8719*, Proteus mirabilis* CECT 8752*, Enterococcus faecalis* CECT 4039*, Staphylococcus aureus* CECT 8753*, Bacillus subtilis* CECT 461 and *Kocuria varians* CECT 230*.* A 0.5 McFarland suspension was prepared from each microorganism and 100 $\mathrm{\mu L}$ were sown in TSA (Ref: CM0131, Oxoid Ltd, United Kingdom).

The *L. plantarum* strain was seeded homogeneously and holes were made using a sterile biopsy punch and placed in the TSA plates in two different ways: one bacteria-bacteria and the other bacteria-agar.

### Evaluation of cryoprotectants

After completing the studies to check the probiotic potential of the strain, it was preserved by lyophilization, a process that requires the addition of a cryoprotectant. For this reason, five different cryoprotectants were evaluated.

Two 1800 mL flasks of MRS broth were prepared. To each flask, 3 mL of the MRS broth in which the porous beads had been resuspended was added. The flasks were incubated at 37 °C for 24 h in an aerobic environment.

The cryoprotectants tested were the following:

- 10% skimmed milk
- Sucrose (Ref: T0167-25G, Sigma-Aldrich Co., USA) 5%
- Skimmed milk 15% + Sucrose 4%
- Trehalose (Ref: T0167-25G, Sigma-Aldrich Co., USA) 15%
- 11% Maltodextrin (Ref: A4804, 1000, PanReac Química S.A., Spain)

Once the cultures were obtained in MRS broth, they were processed to carry out the freeze-drying process. The methodology was the following:

- Distribute 150 mL of the culture in sterile Falcon tubes
- Centrifuge at 4000 rpm for 15 min
- Remove the supernatant and homogenize the pellet with 30 mL of the indicated cryoprotectant in a sterile container
- Freeze the samples for at least 6 h at − 20 °C

This process was repeated until the broth is exhausted. Four pellets were homogenized with the same cryoprotectant to obtain a quantity of product and thus be able to evaluate its effects over time.

Once the freezing time had elapsed, holes were made in the lids of the pots so that they could eliminate the solvent when freeze-dried, and immediately afterwards they were placed in the lyophilizer at a temperature of – 50 °C and a pressure of 0.06 mbar. The freeze-drying time varies between 72 and 96 h, as it ends once the pressure is stabilized around 0.080 mbar.

Subsequently, the 4 pots corresponding to the same cryoprotectant were mixed and added in the same Stomacher bag previously labelled with the name of the corresponding cryoprotectant.

The different products were stored in the refrigerator at 4 °C until the end of the study.

At the time of analysis, 1 g of each product was weighed to make a bank of decimal dilutions up to the 10^–8^ concentration and the concentrations from 10^–6^ to 10^–8^ were seeded on MRS agar. To have more statistical weight, each dilution was seeded in triplicate. These plates were incubated for 24 h at 37 °C in an aerobic environment.

After the incubation time, the dilution plates that presented between 30 and 300 CFU/mL were counted and the average of the three replicates was done to obtain an average value that is the one used to apply the logarithm of the CFU/mL formula.

CFU/mL = $\frac{{{\text{Average}}\;{\text{no}}\;{\text{of}}\;{\text{colonies}}}}{{0.1\;{\text{mL}} \times {\text{Dilution}}\;{\text{in}}\;{\text{which}}\;{\text{we}}\;{\text{count}}}}$; the 0.1 refers to the amount we inoculate into the plate.

This process is repeated every 15 days during the 3 months.

## Results

The results of the phenotypic tests are summarized in the table (see Table 1).

**Table 1:** Summary of phenotypic test results.

Phenotypic tests	Results
Gram stain	Grampositive bacilli
Spores stain	Negative
Catalase	Negative
Oxidase	Negative
Hemolytic capacity	Negative ($\gamma$-hemolysis)
API 50 CH	99.1% *L. plantarum*

### Whole genome sequencing

WIMP platform identified the isolated as *Lactobacillus plantarum*. Finally, the average nucleotide identity (ANI) between our raw assembly and *Lactiplantibacillus plantarum* NCBI Reference Sequence (NZ_CP030105.1) was 99.06%.

### Tolerance to bile salts and lysozyme

**Figure 2:**
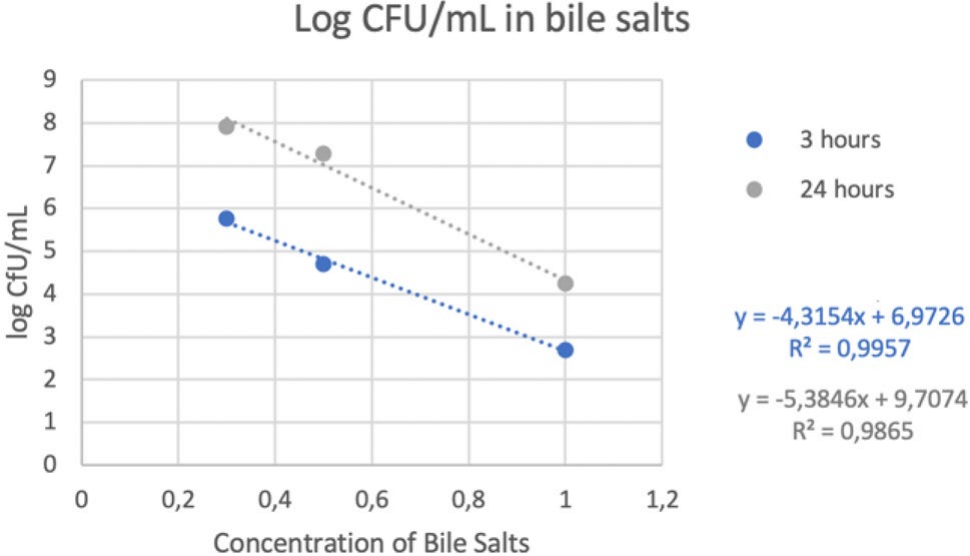
Logarithm of CFU/mL in different concentration of bile salts.

**Figure 3:**
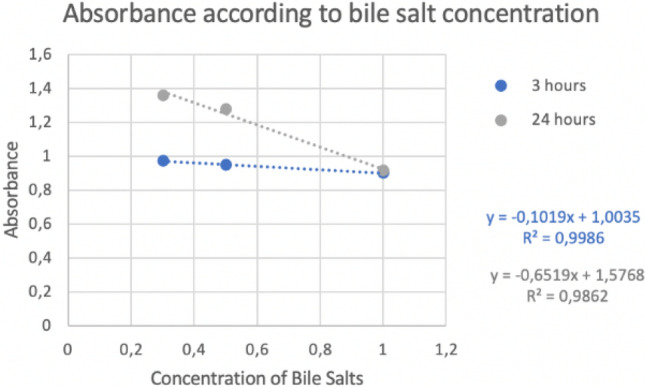
Absorbance in different concentration of bile salts.

**Figure 4:**
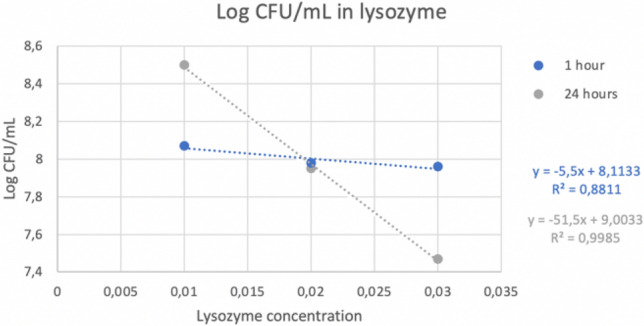
Logarithm of CFU/mL in different concentration of lysozyme.

**Figure 5:**
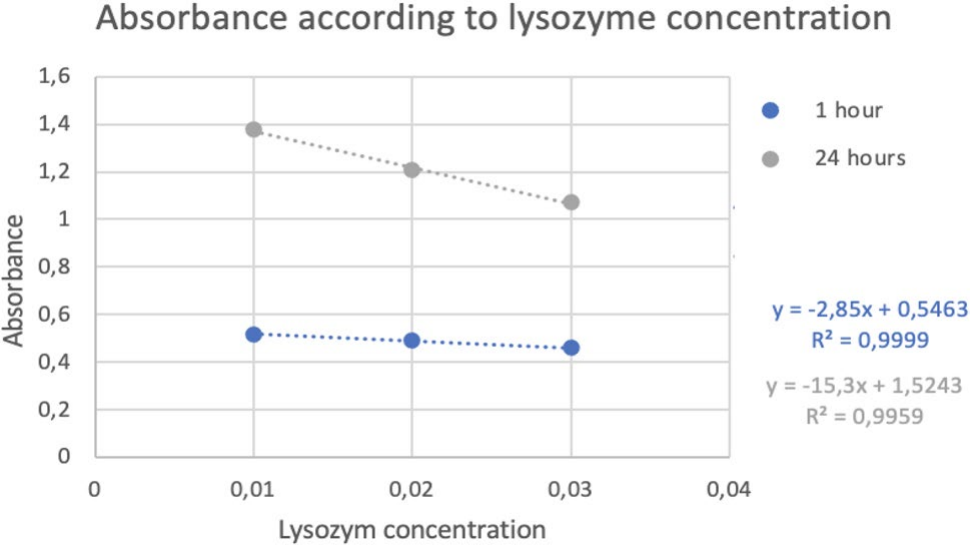
Absorbance in different concentration of lysozyme.

The results of bile salt and lysozyme tolerance in both cases (the logarithm of CFU/mL and the absorbance) are expressed by the next graphs with the trendline and the R value (see Figs. 2, 3, 4, and 5).

### Antimicrobial activity

The antimicrobial activity of our strain is measured using the millimeters of the inhibition halo, for this reason, the results are expressed in millimeters (see Table 2).

**Table 2:** Millimeters of the inhibition halo depending on whether it is in direct contact with the bacteria or the agar.

	Contact with bacteria	Contact with agar
*Pseudomonas aeruginosa*	16	15
*Listeria monocytogenes*	12	10
*Klebsiella oxytoca*	9.5	9
*Escherichia coli*	10.5	10
*Proteus mirabilis*	9	7.5
*Salmonella enterica*	8	8
*Staphylococcus aureus*	9	7.5
*Bacillus subtilis*	9	8
*Kocuria varians*	9	9
*Enterococcus faecalis*	26	22.5

### Antibiotic sesitivity

Antibiotic sensitivity is evaluated with two different tests. In the case of disk diffusion, the millimeters of the inhibition halo are measured and, because four replicates have been made, the 4 values are represented together with the mean (see Table 3), while in the broth dilution method the ELISA reader gives us the absorbance values and is emphasized in bold when bacterial growth begins (see Table 4).

**Table 3:** Disk diffusion method with the millimeters values and the average of different antibiotics.

Antibiotic	Milimeters	Average
Chloramphenicol	22, 21, 22, 23	22
Tetracycline	18′8, 19, 18′7, 19′75	19.063
Ampicillin	19, 15′5, 19′5, 15	17.25
Erythromycin	22, 27, 23, 21′5	23.375
Clindamycin	18′5, 19′2, 20, 18′7	19.1
Kanamycin	23′5, 24, 21, 18	21.625
Gentamicin	22′5, 19′5, 21, 20′7	20.925

**Table 4:** Absorbance in the broth dilution method.

	1	2	3	4	5	6	7	8	9	10	11	12
Clindamycin	0.0898	0.0945	0.0927	0.0952	0.0923	**0**.**1084**	0.1733	0.1459	0.1943	0.2084	0.1958	0.0952
Erythromycin	0.0993	0.1045	0.098	0.098	0.095	**0**.**1142**	0.2018	0.4563	0.1987	0.2022	0.1786	0.1067
Ampicillin	0.0986	**0**.**1526**	0.205	0.186	0.1916	0.1874	0.2342	0.2031	0.2022	0.2541	0.1816	0.0992
Chloramphenicol	0.0993	**0**.**1137**	0.135	0.2111	0.1906	0.1419	0.2063	0.1886	0.2143	0.1889	0.1897	0.0995
Gentamicin	0.1032	0.1055	0.0922	0.0975	**0**.**1057**	0.1063	0.1128	0.1399	0.1897	0.1797	0.1821	0.1056
Tetracycline	0.1193	0.1037	0.1274	0.1414	0.1526	0.1477	0.1453	0.1864	0.1791	0.2082	0.1986	0.1239
Kanamycin	0.0984	0.1026	0.1247	0.1273	0.158	0.1969	0.1934	0.1949	0.228	0.2046	0.2561	0.1048
Blank sample	0.0311	0.0312	0.0296	0.0297	0.0339	0.0317	0.0349	0.0318	0.0318	0.0311	0.0323	0.0335

Boldface marks the value where growth is already detected

### Evaluation of cryoprotectants

**Figure 6:**
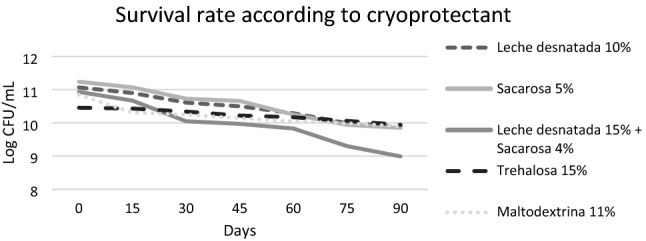
Survival rate depending on cryoprotectant.

The results of the survival rate are expressed in a table with the logarithm of the CFU/mL over time and statistical measures (see Table 5), and the survival rate over time are summarized in a graph (see Fig. 6).

**Table 5:** Values of the analyses over time in the form of logarithm, survival rate and statistical measures.

Cryoprotectants/days	0	15	30	45	60	75	90	Survival rate
Skim milk 10%	11.068	10.9	10.61	10.5	10.29	9.99	9.88	89.2663535
Sucrose 5%	11.24	11.07	10.73	10.66	10.25	9.94	9.85	87.633452
Skim milk 10% + Sucrose 4%	10.934	10.67	10.04	9.97	9.83	9.3	8.99	82.2205963
Trehalose 15%	10.456	10.43	10.34	10.22	10.17	10.06	9.94	95.0650344
Maltodextrin 11%	10.845	10.31	10.25	10.14	10.05	9.99	9.9	91.2863071
Average	10.908	10.676	10.395	10.298	10.118	9.856	9.712	89.0943486
Standard deviation	0.29358	0.31600	0.27486	0.27860	0.18525	0.31373	0.40493	4.73988519
Count	5	5	5	5	5	5	5	5
Standard error	0.13129	0.14132	0.12292	0.12459	0.08284	0.14030	0.18109	2.1197411

## Discussion

Phenotypic characteristics suggested that the study strain corresponded to some species of the genus *Lactobacillus*. Once different phenotypic characteristics, an API 50CH was performed along with molecular identification to determine the species. All results indicated that the strain belonged to the species *Lactiplantibacillus plantarum* (= *Lactobacillus plantarum*)^22^.

Owing to it is a lactic acid bacteria candidate probiotic, the assays indicated in the Guidelines were carried out to determine its probiotic potential. First, its hemolytic capacity was analyzed, giving a negative result and, therefore, fulfilling one of the many properties that probiotics must have because this negative result indicates that it is a safe strain due to the fact that it is not capable of digesting blood cells^23,24^.

Other tests that were carried out were the tolerance to bile salts and lysozyme due to both mark the gastrointestinal conditions through which the probiotic will transit and consequently it must survive these conditions. The results obtained indicated that the higher the concentration of bile salts and lysozyme, the lower the survival rate, but even at high concentrations, it was possible to observe the persistence capacity of the strain in these conditions. These results were supported by a coefficient of determination (R2) of 0.9 which means that there is a very good correlation between the logarithm of CFU and the concentration of bile salts or lysozyme (see Figs. 2, 3, 4, and 5).

On the other hand, it has been possible to demonstrate that the strain under study is capable of elaborating and diffusing compounds with antimicrobial capacity since it presents antimicrobial activity against all the microorganisms tested, as can be seen in Table 2, where it can be observed that the strain can avoid the growth of different microorganisms, generating a halo of inhibition where there is no growth and this halo is measured because the larger the of halo means that it has more capacity to inhibit the growth of the other strain.

Another of the properties that it must be presented is the sensitivity to the antibiotics established for this purpose by the EFSA^18^ and, to corroborate this, two different tests have been carried out. Firstly, the disc diffusion method has been carried out by which the inhibition halo has been measured, and the results obtained have been compared with the values established by the company Becton Dickinson summarized in Table 6.

**Table 6:** Values in mm from which indicates sensitivity to these antibiotics.

Antibiotic	Chloramphenicol	Tetracycline	Ampicillin	Erythromycin	Clindamycin	Kanamycin	Gentamicin
Values	> 21	> 19	> 17	> 23	> 19	> 18	> 15

Comparing the values in Table 3 with those in Table 6, it was possible to determine that the strain is sensitive to all antibiotics. Likewise, the broth dilution method was performed to corroborate the sensitivity of the strain to these antibiotics. Continuing with the results represented in Table 4, it can be shown how all the values in column 1 are like those in column 12, being those corresponding to the negative control where only the culture medium was added, indicating that there is no bacterial growth at the maximum concentrations of the corresponding antibiotic marked by the EFSA, denoting the sensitivity of the strain to all the antibiotics tested.

With all this, it can be affirmed that strain of *L. plantarum* can be administered as a probiotic because it meets the different requirements established by the FAO and the WHO^1^. In addition, the results obtained agree with the results obtained by similar studies^15,16,20^.

The main objective of this study, as already indicated, is to establish whether the strain isolated from the intestinal microbiota of *Helix aspersa* Müller meets the requirements that allow it to be added to the feed of these animals and thus be able to improve their health status. Therefore, due to the positive results, different cryoprotectants are studied to evaluate which of them allows the strain a higher survival rate over time. The results represented in Table 5 indicate that the most effective cryoprotectant is 15% trehalose with a 95% survival rate after 90 days. In addition, Fig. 6 also shows that it is the most stable cryoprotectant because of the concentration of the probiotic in suspension in this cryoprotectant does not show a significant decrease over the time evaluated. Therefore, the best cryoprotectant is a disaccharide^11^.

## References

[1] FAO/WHO: Health and nutritional properties of probiotics in food including powder milk with live lactic acid bacteria. (2001). www.fao.org. Accessed Apr 2021.

[2] Hassan MU, Nayab H, Shafique F, Williamson MP, Almansouri TS, Asim N, Shafi N, Attacha S, Khalid M, Ali N, Akbar N (2020). Probiotic properties of *Lactobacillus helveticus* and *Lactobacillus plantarum* isolated from traditional Pakistani yoghurt. Biomed. Res. Int..

[3] Heyman M, Ménard S (2002). Probiotic microorganisms: How they affect intestinal pathophysiology. Cell. Mol. Life Sci..

[4] FAO/WHO. Probiotics in food: Health and nutritional properties and guidelines for evaluation. FAO Food and Nutrition paper 85 (2006).

[5] Peivasteh-Roudsari L, Pirhadi M, Karami H, Tajdar-Oranj B, Molaee-Aghaee E, Sadighara P (2019). Probiotics and food safety: An evidence-based review. J. Food Hyg. Saf..

[6] Ganguly NK, Bhattacharya SK, Sesikeran B, Nair GB, Ramakrishna BS, Sachdev HPS, Batish VK, Kanagasabapathy AS, Muthuswamy V, Kathuria SC, Katoch VM, Satyanarayana K, Toteja GS, Rahi M, Rao S, Bhan MK, Kapur R, Hemalatha R (2011). ICMR-DBT guidelines for evaluation of probiotics in food. Indian J. Med. Res..

[7] Weese JS, Martin H (2011). Assessment of commercial probiotic bacterial contents and label accuracy. Can. Vet. J..

[8] García MD, Uruburu F (2000). La conservación de cepas microbianas. Actualidad SEM.

[9] Arencibia D, Rosario L, Gámez R (2008). Métodos generales de conservación de microorganismos.

[10] Li B, Tian F, Liu X, Zhao J, Zhang H, Chen W (2011). Effects of cryoprotectants on viability of *Lactobacillus reuteri* CICC6226. Appl. Microbiol. Biotechnol..

[11] Hubálek Z (2003). Protectants used in the cryopreservation of microorganisms. Criobiology.

[12] Vera, R. E. *Microbiología del caracol Helix aspersa Müller. Aplicaciones biotecnológicas para su mejoramiento sanitario con impacto en su comercialización*. Doctoral thesis, Universidad Autónoma de Barcelona (2016).

[13] Storey BT, Noiles EE, Thompson KA (1998). Comparison of glycerol, other polyols, trehalose, and raffinose to provide a defined cryoprotectant medium for mouse sperm cryopreservation. Cryobiology.

[14] Siaterlis A, Deepika G, Charalampopoulos D (2008). Effect of culture medium and cryoprotectants on the growth and survival of probiotic lactobacilli during freeze drying. Lett. Appl. Microbiol..

[15] Girmé, G. *Caracterització microbiològica i enzimática de la llet d’euga gestant. Avaluació de les propietats probiòtiques, antimicrobianes i preservadores*. Doctoral thesis, Universidad Autónoma de Barcelona (2015).

[16] Sossa DP, González LM, Vanegas MC (2009). Aislamiento e identificación de *Lactobacillus* contaminantes en una planta colombiana de fermentación alcohólica. Revista UDCA Actualidad & Divulgación Científica.

[17] Juul, S. *et al.* What’s in my pot? Real-time species identification on the MinION^TM^. Preprint at bioRxiv: 0307422 (2015).

[18] Kolmogorov M, Yuan J, Lin Y, Pevzner PA (2019). Assembly of long error-prone reads using repeat graphs. Nat. Biotechnol..

[19] Jain C, Rodriguez-R LM, Phillippy AM, Konstantinidis KT (2018). High throughput ANI analysis of 90K prokaryotic genomes reveals clear species boundaries. Nat. Commun..

[20] Vinderola CG, Reinheimer JA (2003). Lactic acid starter and probiotic bacteria: A comparative “in vitro” study of probiotic characteristics and biological barrier resistance. Food Res. Int..

[21] European Food Safety Authority (2012). Guidance on the assessment of bacterial susceptibility to antimicrobials of human and veterinary importance. EFSA J..

[22] List of Prokaryotic names with Standing in Nomenclature. Genus *Lactiplantibacillus*. https://lpsn.dsmz.de/genus/lactiplantibacillus. Accessed May 2021.

[23] Buxton R (2005). Blood Agar Plates and Hemolysis Protocols.

[24] Oh YJ, Jung DS (2015). Evaluation of probiotic properties of *Lactobacillus* and *Pediococcus* strains isolated from *Omegisool*, a traditionally fermented millet alcoholic beverage in Korea. LWT Food Sci. Technol..

